# Applying Characteristic Impedance Compensation Cut-Outs to Full Radio Frequency Chains in Multi-Layer Printed Circuit Board Designs

**DOI:** 10.3390/s24020675

**Published:** 2024-01-21

**Authors:** Vaidotas Barzdenas, Aleksandr Vasjanov

**Affiliations:** Department of Computer Science and Communications Technologies, Vilnius Gediminas Technical University, 10105 Vilnius, Lithuania; aleksandr.vasjanov@vilniustech.lt

**Keywords:** compensation, cut-out, impedance, high frequency, PCB, printed circuit board, RF chain

## Abstract

Modern wireless communication systems are of utmost importance to various sectors such as healthcare, education, the household, and the advancement of emerging technologies like the internet of things, autonomous vehicles, and the enhancement of 5G. Further development and improvement of these systems drives the need for small dimension, high integration and density, and cost-effective electronic devices. Achieving optimal performance in wireless electronic devices involves overcoming engineering challenges related to microstrip line signal integrity. This research addresses the impact of surface mount technology (SMT) component pads on signal integrity, proposing a novel high-frequency microstrip line structure for mitigating impedance discontinuities. The study introduces stepped microstrip lines and explores characteristic impedance compensation techniques. A six-layer printed circuit board (PCB) structure is presented, and the effects of compensation on signal integrity are analyzed using time-domain reflectometry and scattering parameter measurements. The results demonstrate the effectiveness of compensation methods in aligning characteristic impedance with desired values, thereby ensuring improved impedance matching and transmission coefficients. The average over-the-length impedance for the proposed structure with compensation applied was measured to be 52.7 Ω, which is only 1.3 Ω (2.5%) more than that of the reference microstrip. Applying reference plane cut-outs leads to a maximum compensated absolute value of more than 30 Ω to reach the target impedance with a 10% tolerance. This research contributes valuable insights for advancing wireless communication systems and maintaining robustness in high-frequency microstrip transmission lines.

## 1. Introduction

Wireless communication systems have evolved into indispensable components across diverse sectors, impacting domains such as health, education, the internet of things (IoT), smart cities, autonomous vehicles, media and entertainment, manufacturing, Industry 4.0, finance, agriculture, energy, and various other fields [1,2,3]. The application of wireless technologies in these fields is very important for advancing smarter electronic devices that can independently and wirelessly exchange information, execute actions, intelligently control each other, and generate vast amounts of data, thus providing users with an abundance of information [4,5].

The goal of modern wireless electronic devices is to make them as small as possible by creating PCBs with high integration and density that are reliable and long-lasting and, at the same time, achieving the lowest possible production costs. This challenge encapsulates various engineering hurdles, such as implementing RF and digital high-frequency circuits without compromising fundamental parameters, minimizing electromagnetic compatibility (EMC) emissions, and fortifying EMC susceptibility, all while operating under low energy consumption conditions. These engineering challenges drive research on the signal integrity of microstrip lines. When integrating high-speed or high-frequency interconnection solutions into a PCB, even slight disruptions in the physical geometry along the microstrip transmission line can have a substantial impact on the integrity of the transmitted signal. This compromise in signal integrity materializes through diverse manifestations, including additional reflections and impedance changes, loss of signal amplitude, diminished signal rise/fall times, heightened jitter, overshoots/undershoots, and other adverse effects. Ensuring optimal signal transmission necessitates the identification and mitigation of these discontinuities within high-speed or RF channels. It is imperative to address these challenges in order to maintain the robustness and efficiency of a communication system.

The dissimilarity in widths between surface mount technology (SMT) component pads and microstrip transmission lines frequently leads to impedance discontinuities and compromises signal integrity. The broader SMT component pads introduce excessive shunt capacitance, leading to a disruption in the transmission line. These SMT components play a crucial role in various engineering solutions, such as aligning transceiver circuits with antennas, offering common-mode suppression, implementing DC-blocking, providing electrostatic discharge protection, ensuring short-circuit protection, and fulfilling other essential functions. However, the essential inclusion of SMT components inadvertently induces an undesired alteration within transmission line impedance. Addressing this impedance decline necessitates a careful approach to the ground plane region beneath the SMT pads, thus ensuring proper modification to minimize impedance alterations along the signal propagation path.

This paper is organized as follows: the introduction is followed by a description of the motivation for this research and existing compensation methods in Section 2. Section 3 describes the novelty of the proposed approach compared with existing methods. Section 4 describes the test bench and simulation results, while Section 5 contains the measurement results and discussion. The results presented in this paper are then summarized in Section 6, with references provided afterward.

## 2. Motivation and Existing Compensation Methods

The reference plane cut-out compensation technique, sometimes referred to as compensation using a defected ground structure (DGS), has been a topic of research in multiple papers that cover different case studies, such as individual SMA connector pads, track bends, etc. [6,7]. Individual reference plane cut-outs under the discontinuities can be shaped differently. The cut-out can be smaller, larger, or have the same width as the discontinuity. This has been reported in various papers and design notes [8,9,10,11,12], but they are only single case studies addressing passive surface-mount device components such as 0201 or 0402 package size capacitors. Moreover, impedance compensation techniques, including reference plane cut-out introduction, have been thoroughly analyzed and improvements have been proposed in previous papers [13,14] on the latter topic.

In addition, there are scholarly works addressing the challenge of high bond wire interconnect inductance. This is achieved through the incorporation of a grounding pad in close proximity to the connection and the application of SMD capacitors [15,16,17]. This integration introduces supplementary capacitance into the RF chain, effectively mitigating overall impedance along the transmission line. Furthermore, the scientific literature offers valuable insights into strategies for compensating impedance discontinuities in RF SMP connectors [18]. The impedance of the RF tract at the SMP connector is harmonized by narrowing the width of the microstrip line in the welding area. Additionally, it is recommended to shorten the microstrip line in the direction of the energy transmission. The implementation of these techniques not only addresses impedance discrepancies but also significantly improves the performance and efficiency of SMP connectors. There are scientific publications as well as engineering recommendations that provide methods for compensating impedance discontinuities in SMA edge type connectors [6,19]. The best practices involve incorporating cut-outs into ground reference layers, compensating with coplanar structures, and employing pad taper for optimal connector performance. 

A discontinuity of a different impedance appears in a microstrip when one or more components connected to it has a land pad wider than the width of the microstrip. If the discontinuity and microstrip width ratio is small (ex. < 2) and the number of discontinuities is few, then the negative effect (reflections resulting in higher losses) can be neglected. On the other hand, when a microstrip contains multiple large discontinuities, this will lead to heavy losses in signal amplitude, which are frequency-dependent. If the discontinuity is larger than *λ*/4, then reflections will occur, and the drop in amplitude will be seen in the microstrip insertion loss *S*_21_ parameter. This is because each discontinuity presents a parasitic capacitance loading the microstrip and thus changing the desired characteristic impedance. Moreover, the larger the area of the discontinuity is the larger the capacitance becomes, and the impedance becomes smaller according to Equation (1) [20,21,22].

(1) $$Z_{0}=\sqrt{\frac{R+j\omega L}{G+j\omega C}}={\left.\sqrt{\frac{L}{C}}\right|}_{R=0;G=0},$$

where *R* is the series resistance per unit length (Ω/m), *L* is the series inductance (H/m), *G* is the shunt conductance (Ʊ/m), and *C* is the shunt capacitance (F/m). For an ideal lossless line, which is considered in this simplified analysis, the parasitic series resistance, *R*, and the parasitic parallel conductance, *G*, are equal to zero.

**Figure 1:**
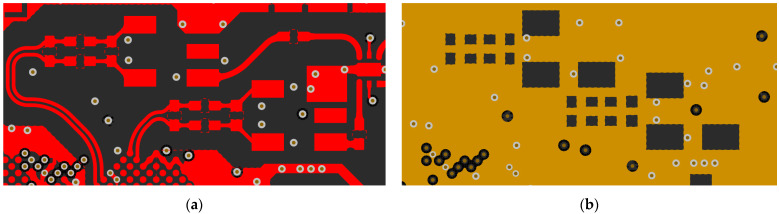
Reference plane cut-out impedance compensation practical application as follows: (**a**) top copper layer containing microstrips with discontinuities and (**b**) second layer reference plane compensation cut-outs.

Multilayer printed circuit boards (PCBs) can contain multiple reference (ground) layers, so the capacitance of each discontinuity can be changed by means of creating cut-outs in the reference layer directly beneath it. An example of that is shown in Figure 1. Figure 1a presents differential traces with an impedance of 100 Ω connected to *π*-type impedance matching network components, encapsulated in a 0402 SMT package. The impedance matching network is then connected to a high-frequency transformer for single-ended to differential conversion. It can be clearly seen that the component pads in the described RF chain are wider than the differential traces. If no action is taken, these pads create discontinuities that attenuate the signal. In order to reduce the added parasitic capacitance, reference plane cutouts, shown in Figure 1b, are introduced in the second copper layer. Thus, the third copper layer becomes the reference layer for these pads. Different stack-ups have been thoroughly analyzed in [14], while obtaining the optimal size of the cutout has been discussed in [13].

While individual reference plane cut-outs under each discontinuity do indeed solve the discontinuity-inflicted impedance change issue, applying them in practical designs is not that straightforward. The reference planes have to be connected with the least possible impedance, and, ideally, a metallized via interconnect has to be present in close vicinity to each cut-out. The reason for this is that the return current has to take the path of least impedance [23,24]. A change in reference planes occurs when implementing impedance compensation cut-out techniques, so these planes have to be connected for the signal not to be distorted. As a result, when multiple reference plane cut-outs are introduced, a lot of additional metallized vias have to be included in close proximity to each cut-out. This is not the case in Figure 1, not because it is a design flaw but due to the area availability and price. Metallized vias running through all the layers can affect routing on other internal layers, which is not a desirable outcome in dense designs. On the other hand, adding blind vias only for this purpose will greatly increase the manufacturing price, as will the larger overall number of vias on the PCB. All in all, impedance compensation through applying reference plane cut-outs could be improved for better design for manufacture (DFM) compliance.

## 3. Novelty

Published papers on the topic of discontinuity impedance compensation using reference plane cut-outs suggest adding individual impedance cut-outs under each discontinuity. This paper, on the other hand, proposes altering the microstrip width, which connects the discontinuities, to the width of the latter and applying a cut-out to the whole section. If there are multiple discontinuity widths in the RF chain, the same principle is applied to each width.

**Figure 2:**
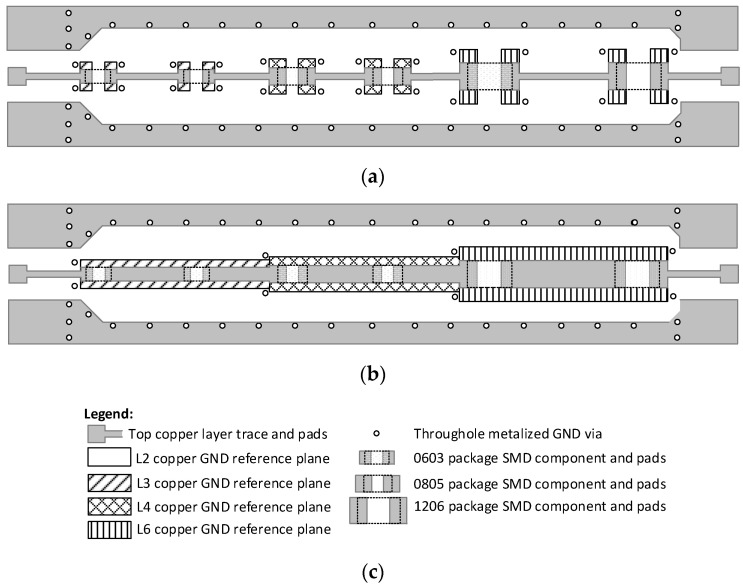
Microstrip impedance discontinuity compensation (**a**) using separate reference plane cut-outs, which is an existing method, (**b**) a proposed method of changing the width of the microstrip and forming cut-outs for different width segments, and (**c**) legend.

As a result, the designer has two possible paths, described in Figure 2, to choose from when applying cut-outs for mitigating microstrip characteristic impedance discontinuities in multilayer PCBs. 

The first one, shown in Figure 2a, suggests adding a required amount of separate reference plane cutouts under each discontinuity. In this case, microstrip section widths, which connect the discontinuities, are left unchanged. A different design strategy, which is proposed in this paper (Figure 2b), suggests altering the width of the microstrip sections that connect the discontinuities to the width of the latter ones. Additionally, reference plane cutouts are introduced under each segment of the same width. The rule of thumb for the optimal reference plane cut-out width is that it is two times larger than the width of the discontinuity [6] and the cut-outs can be applied to PCB stackups with different dielectrics [14]. Figure 2c is the legend for Figure 2a,b. A summary of existing impedance discontinuity compensation techniques is presented in Table 1.

## 4. Test Bench Setup and Simulation

This paper proposes altering the existing microstrip width to a certain discontinuity width and applying reference plane cut-out compensation to the latter segment. As a result, a stepped-width microstrip is formed, but the impedance is held constant by removing reference plane copper directly under each step segment.

**Figure 3:**
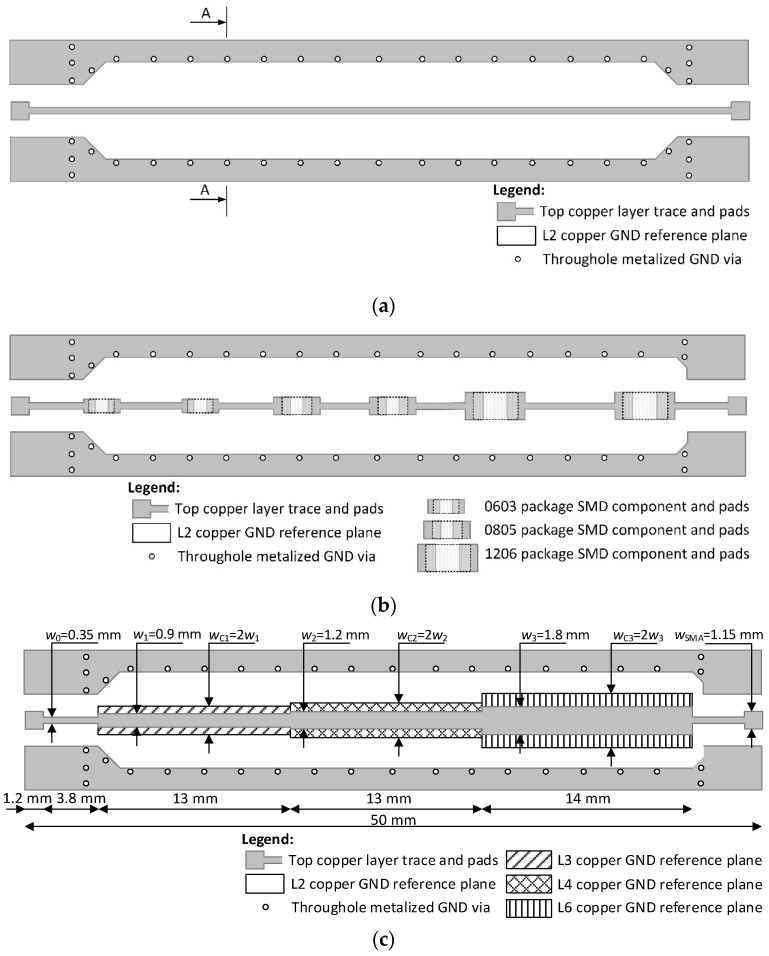
Microstrip with and without discontinuity configurations as follows: (**a**) reference 50 Ω microstrip, (**b**) 50 Ω microstrip with different size components, and (**c**) modified microstrip with impedance compensation.

Figure 3a presents a top view of the structure of the high-frequency microstrip line with a second ground reference layer formed in the upper (first) copper layer. This microstrip line serves as a reference for measurements, which are considered desirable and are compared with the measurement results of the proposed high-frequency microstrip line structure. The characteristic impedance (*Z*_0_) of the microstrip line is set to be 50 Ω. Soldering pads at the ends of the line are used to connect a high-frequency SMA connector to the microstrip line. In the upper (first) copper layer, there is also a ground reference plane formed, which is connected to other reference ground planes formed in other conductive layers.

In order to assess the impact of SMD components and to quantitatively and qualitatively evaluate the parameters of such a line, as well as to compare them with the measurements of the line presented in Figure 3a, a stepped microstrip line is formed in Figure 3b. The entire ground reference layer in Figure 3b corresponds to the second layer of the structure. Additionally, no impedance compensation is applied to any segment of the microstrip line in this high-frequency microstrip line. The goal is to make the characteristic impedance of this high-frequency microstrip line equal to or close to *Z*_0_, but all its segments, which have a width greater than that of the reference *Z*_0_ microstrip line, will have a lower characteristic impedance, consequently increasing the power losses of the transmitted signal.

Meanwhile, in Figure 3c, a high-frequency trace is presented in which characteristic impedance compensation is applied to all segments wider than the *Z*_0_ characteristic impedance line. In this figure, the narrowest line segment has an eliminated copper region in the second conductive layer, and the ground reference copper layer of this segment becomes the third conductive layer. A line segment with a medium width has an eliminated ground reference copper region in the second and third conductive layers, and the ground reference copper layer for these segments is the fourth conductive layer. It should be noted that when characteristic impedance compensation is applied, it is necessary to connect the ground reference copper planes of different layers with as many vias as close as possible to the compensated segment. The characteristic impedance of the line segment with a medium width is brought closer to the desired *Z*_0_ by utilizing compensation with two ground reference copper layers. Meanwhile, the widest line segment eliminates the ground reference copper areas in the second, third, fourth, and fifth conductive layers, and the ground reference copper layer for these segments is the sixth (bottom) conductive layer.

**Figure 4:**
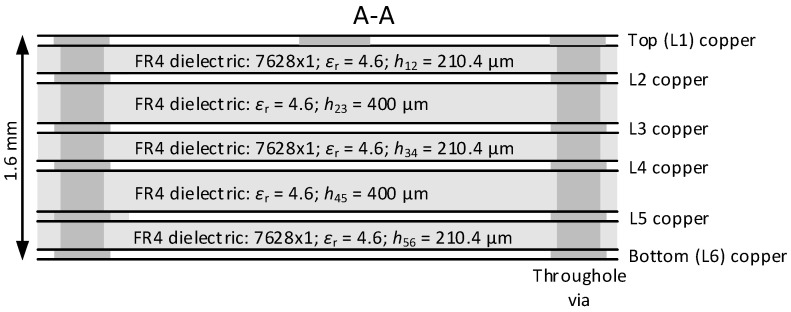
Standard 1.6 mm six-layer PCB stackup and thickness for JLC06161H-7628 [25].

Figure 4 shows a cross-section (cut A-A) of a high-frequency tract microstrip line stand printed circuit board structure. The printed circuit board consists of six conductive copper layers, where the thickness of the outer layers is 35 μm and the thickness of the inner layers is 15.2 μm. The conductive copper layers are interconnected through an FR4-type dielectric material, with a dielectric constant of *ε*_r_ = 4.6 and a respective thickness of 210.4 μm and 400 μm. Different copper layers are connected to each other using metallized vias with a diameter of 0.3 mm. The total thickness of the high-frequency microstrip line stand printed circuit board is 1.6 mm.

**Figure 5:**
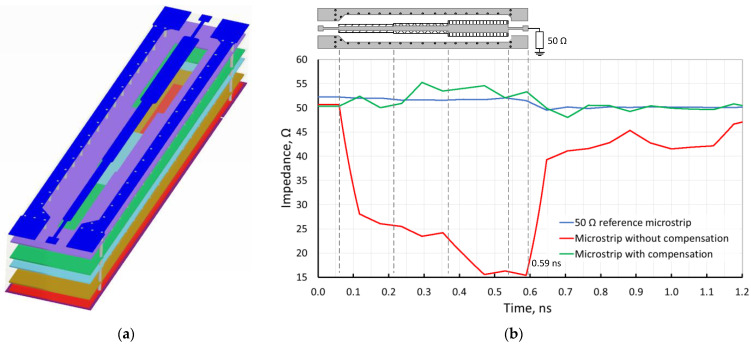
Proposed compensated RF chain. (**a**) Three-dimensional model and (**b**) TDR simulation results.

A six-layer model, based on the discussion above, has been created using Keysight’s ADS EM simulation tool, which is presented in Figure 5a. Different layer colors highlight all six model PCB copper layers and help distinguish the reference layer to each microstrip segment on the top (blue). The simulation results of this structure are given in Figure 5b. The simulation results revealed the possibility of maintaining a desired characteristic impedance by altering the width of the microstrip to match the width of individual discontinuities and carefully selecting the appropriate reference layer in a multi-layer PCB.

## 5. Measurement Results and Discussion

**Figure 6:**
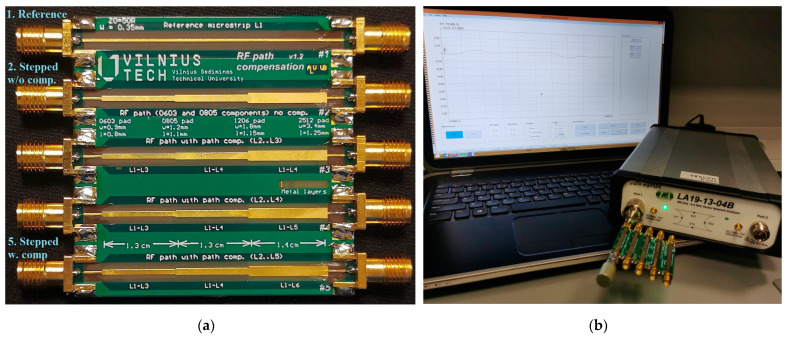
Device under test. (**a**) Printed circuit board and (**b**) measurement test bench.

The measurements were conducted using an open-short-load (OSL)-calibrated LA19-1304B VNA. The device under test (DUT), which is presented in Figure 6a, was connected directly to the VNA avoiding the use of additional cables, as shown in Figure 6b. The DUT, which is presented in Figure 6a, contains five separate circuits. The first of these circuits is the reference 50 Ω microstrip line. The second circuit corresponds to the stepped microstrip line, which meets 0603 and 0805 size surface mount technology (SMT) component land pads and does not include cut-outs in the reference plane under the wider segments. Circuits three and four present intermediate results, containing cut-outs in the reference plane under the wider segments up to and including copper layer three (L3) and four (L4) accordingly. As expected, they are not mentioned in the paper due to the results not satisfying the target overall impedance of 50 Ω. Finally, the fifth circuit in the DUT board contains reference planes on copper layers L3, L4 and L6 for each of the width steps and corresponds to the final structure that meets the target impedance.

**Figure 7:**
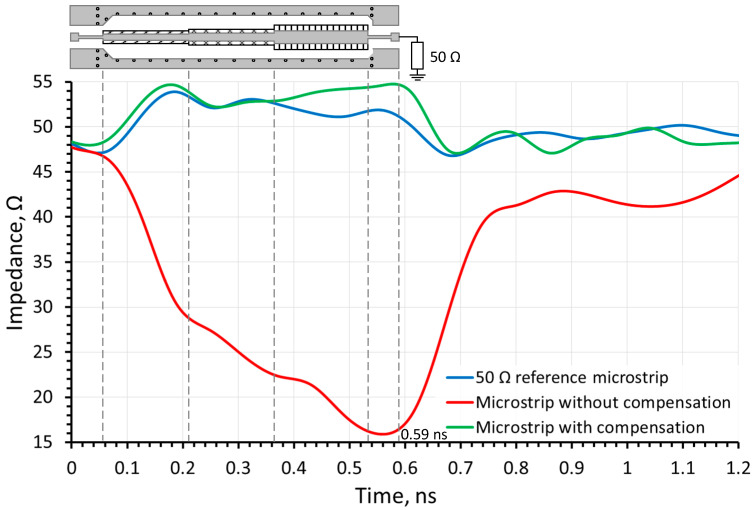
TDR measurement results.

Figure 7 depicts the results of the time-domain reflectometry measurements for the sample structure. In this figure, the *x*-axis represents the propagation time of the electrical signal, while the *y*-axis indicates the characteristic impedance of the high-frequency microstrip line. The regions on the time-domain reflectometry measurement graphs corresponding to the characteristic impedance of the segments of the high-frequency tract are also indicated in Figure 7. The time it takes for the TDR signal to reach the load is 0.59 ns according to Equation (2):

(2) $$t=\frac{2l}{v_{m}}=\frac{2l\sqrt{\varepsilon_{eff}}}{c}$$

where *ε_eff_* ≈ 3.1 [26,27] is the effective dielectric constant, *l* = 50 mm is the length of the DUT and, because the incident wave from the VNA has to travel to the load and back, the length is doubled. The latter timestamp is marked in the TDR simulation and measurement plots (Figure 5b and Figure 7) and is held to be the end of the line.

The dashed black curve corresponds to the reflectometry measurement results of the reference microstrip line structure loaded with a high-frequency 50 Ω calibration load. These results serve as a reference, meaning they are compared with subsequent measurements of the same type. As can be seen from this figure, which closely correlates to the simulation results in Figure 5, the characteristic impedance *Z*_0_ measures approximately 50 Ω, with a guaranteed tolerance provided by the manufacturers of up to 10% from the nominal value. The average measured reference microstrip impedance value from the start to the end (0.59 ns) is 51.4 Ω. The solid gray curve represents the time-domain reflectometry measurements for high-frequency microstrip lines of varying widths without any compensation applied. Deviations in characteristic impedance from the reference measurements reach a minimum of 15.9 Ω at the widest point of the microstrip line. 

Furthermore, due to the significant deviation from the desired *Z*_0_ = 50 Ω value, signs of a high-frequency tract mismatch are observed beyond 0.7 ns, although it is a fully matched load. This leads to a conclusion that the reflections in the microstrip with very large discontinuities can affect the way the load impedance is seen by the source, even if it is correctly matched. In this case, the average impedance value over the whole length is around 28.2 Ω. The solid black curve corresponds to the time-domain reflectometry measurement results of high-frequency microstrip lines with different widths where compensation has been applied. Based on the obtained results, the applied compensation effectively altered characteristic impedance values that closely align with the reference measurements, deviating by no more than 10% from the target *Z*_0_ = 50 Ω with an acceptable level of tolerance. The average measured over-the-length impedance for the latter is around 52.7 Ω, which is only 1.3 Ω (2.5%) more than that of the reference microstrip.

**Figure 8:**
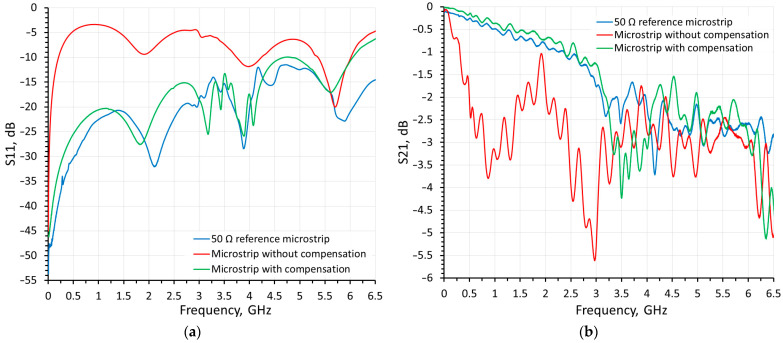
*S*-parameter measurement results of (**a**) *S*_11_ and (**b**) *S*_21_.

The results of frequency-domain scattering parameter measurements for the test structure are presented in Figure 8. In these figures, the *x*-axis corresponds to the electrical signal frequency, while the *y*-axis indicates the quality of impedance matching (*S*_11_) and the transmission coefficient (*S*_21_) of the high frequency microstrip line. Both graphs have their y-axes specified in relative decibel units. The dashed black curve in Figure 8a represents the impedance matching quality (*S*_11_ measurements) of the reference microstrip line. This curve serves as a reference against which further measurements of this type are compared. This graph indicates impedance matching quality better than −10 dB from 2 MHz to 6.5 GHz. The solid gray curve in Figure 8a corresponds to the impedance matching quality (*S*_11_ measurements) of high frequency microstrip lines with different widths but without any compensation applied. According to this curve, the impedance matching quality is less than −3 dB across the entire frequency range and is worse compared with the reference curve. The solid black curve in Figure 8a corresponds to the impedance matching quality (*S*_11_ measurements) of high-frequency microstrip with applied compensation. The observed impedance matching is close to the reference *S*_11_ measurements and demonstrate the effectiveness of the compensation method used.

The dashed black curve in Figure 8b represents the transmission coefficient (*S*_21_ measurements) of the reference microstrip line structure. This curve serves as a reference for further measurements of this type. The solid gray curve in Figure 8b corresponds to the transmission coefficient (*S*_21_ measurements) of high-frequency microstrip lines with different widths without any compensation applied. It is important to note a reduced transmission coefficient in the frequency range compared with the reference *S*_21_ measurements curve, thereby indicating higher losses in the high-frequency path. The solid black curve corresponds to the transmission coefficient (*S*_21_ measurements) of high-frequency microstrip lines with applied compensation. The presented transmission characteristic closely resembles the reference *S*_21_ measurements, thus confirming the efficiency of the compensation method.

The measurement results confirm the possibility of altering the initial microstrip line width to the width of the consecutive components that are connected to it without affecting the performance. The final structure is deprived of discontinuities and takes the shape of a stepped microstrip with appropriate impedance compensation cut-outs under each segment. The latter structure could be applied to both high- and low-power applications, such as power amplifier (PA) chains. In the case of low-power and dense applications, such as portable wireless devices, the size and the diversity of the components connected to the microstrip are little, so the number of width changes could be one or two. These devices usually employ passive component SMD packages in a size range from 01005 to 0402. In high-power applications, such as base station PAs, the devices (transistors, isolators, filters, etc.) have to cope with watt-level power and are thus physically large and require large solder pads. The intermediate pre-amplification stage in a high-power base station can contain components in the range of 0603 to 0805 SMD device sizes, thus resulting in one compensated microstrip step size, whereas the power stage with even larger packages results in a different compensated microstrip step size.

## 6. Conclusions

The proposed high-frequency microstrip line structure, which includes stepped microstrip lines and characteristic impedance compensation techniques, was put into practice and subjected to thorough analysis on a six-layer printed circuit board (PCB). The central focus of the study was to evaluate the impact of compensation on signal integrity using time-domain reflectometry and scattering parameter measurements. The findings unequivocally highlight the effectiveness of the compensation methods in aligning characteristic impedance with desired values, thereby ensuring enhanced impedance matching and transmission coefficients. The average over-the-length impedance for the proposed structure with compensation applied was measured to be 52.7 Ω, which is only 1.3 Ω (2.5%) more than that of the reference microstrip and is well within the current industry standard level of tolerance. Applying reference plane cut-outs lead to a maximum compensated absolute value of more than 30 Ω to reach the target impedance. These results emphasize the practical viability of the approach and provide valuable insights for the advancement of wireless communication systems and maintaining signal integrity in high-frequency microstrip transmission lines.

## Figures and Tables

**Table 1:** Impedance discontinuity compensation technique summary.

Specification/Requirement	Impedance Discontinuity Compensation Technique			
	Taper	Bond wire/Capacitor	Separate Cut-Out	Proposed
Minimal number of PCB layers required	2	2	4	4
Additional PCB elements required	–	Lumped components, bond wires	Through connecting different reference planes in close proximity to each cut-out	
PCB area	Large for wideband operation	Medium	Low	Low
Complexity	Low	High	Medium	Medium
Price	Low/Medium	High	Medium	Medium
Remark	Large bandwidth is very hard to achieve, especially for dense layouts with multiple discontinuities	Additional lumped components and bond wires.	–	Reduced number of reference plane interconnect vias required compared with separate cut-outs. Microstrip width can be adjusted to achieve precise *Z*_0_ with a specific stackup

## Data Availability

Data are contained within the article.
